# Tuning the decay of sound in a viscous metamaterial

**DOI:** 10.1098/rsta.2022.0007

**Published:** 2022-11-28

**Authors:** M. Ibarias, J. Doporto, A. A. Krokhin, J. Arriaga

**Affiliations:** ^1^ Instituto de Física, Universidad Autónoma de Puebla, Apartado Postal J-48, 72570 Puebla, México; ^2^ Department of Physics, University of North Texas, 1155 Union Circle #311427, Denton, TX 76203, USA

**Keywords:** phononic crystals, homogenization, viscosity

## Abstract

Using analytical results for viscous dissipation in phononic crystals, we calculate the decay coefficient of a sound wave propagating at low frequencies through a two-dimensional phononic crystal with a viscous fluid background. It is demonstrated that the effective acoustic viscosity of the phononic crystal may exceed by two to four orders of magnitude the natural hydrodynamic viscosity of the background fluid. Moreover, the decay coefficient exhibits dependence on the direction of propagation; that is, a homogenized phononic crystal behaves like an anisotropic viscous fluid. Strong dependence on the filling fraction of solid scatterers offers the possibility of tuning the dissipative decay length of sound, which is an important characteristic of any acoustic device.

This article is part of the theme issue ‘Wave generation and transmission in multi-scale complex media and structured metamaterials (part 2)’.

## 1. Introduction

Dissipation accompanies propagation of sound in any elastic medium, leading to exponential decay of sound waves with distance. In a homogeneous fluid of density ρ, the sound amplitude decays approximately as $e^{-\gamma_{0}x}$, where $\gamma_{0}$ is the decay coefficient. For a monochromatic plane wave, the decay coefficient $\gamma_{0}=\omega^{2}(4\eta /3+\xi )/(2\rho c^{3})$ grows with frequency as $\omega^{2}$, depending on the speed of sound c, and it is linear with respect to the viscosity coefficients η and ξ [1]. For frequencies around 100 kHz, the propagation length $1/\gamma_{0}$ of sound in water is several kilometres. Therefore, viscous losses in the bulk can be neglected in the design of devices of sizes a few metres or centimetres. However, when a sound wave meets a solid boundary, a narrow viscous layer of thickness $\delta ={\left(2\eta /(\omega \rho )\right)}^{1/2}$ is formed. Velocity gradients within this viscous layer greatly exceed the gradients in the bulk, leading to much higher viscous losses than in free fluid [2,3]. Moreover, if the sound wave meets a set of solid boundaries, multiple reflections and viscous friction in narrow channels strongly increase energy losses.

In the case of a periodic distribution of scatterers, dissipation becomes a dominant factor that defines the efficiency of any acoustic device [4–14]. In particular, double-negative behaviour of a well-designed dissipationless phononic crystal can be completely suppressed owing to high viscous losses. Enhanced dissipation may strongly reduce the engineering metamaterial properties, in particular the effect of negative refraction [15], hydrodynamic cloacking [16] and the sensitivity of tubular phononic crystal sensors [17].

In some cases, viscous losses are desirable in devices that reduce external noise. Modern sound absorbers use innovative designs based on metamaterials. Artificial acoustic metamaterials can be used as structures to increase sound absorption to extents not achieved in natural materials [18,19]. In phononic metamaterials containing hard scatterers in a fluid background, viscous dissipation breaks the time-reversal symmetry, making the fluid dynamics irreversible and even non-reciprocal if mirror symmetry is also broken [20,21].

In the presence of solid scatterers, the energy of a sound wave propagating in a solid–fluid structure is dissipated mainly within the narrow boundary layer δ formed over each scatterer. Therefore, dissipation in the bulk fluid with the rate $\gamma_{0}$ can be neglected, whicht yields $\gamma_{0}\ll \gamma_{\mathrm{ph}}$, where $\gamma_{\mathrm{ph}}$ is the decay coefficient within the phononic crystal. At the same time, the decay coefficient $\gamma_{\mathrm{ph}}$ should be sufficiently weak to allow propagation over many periods $a_{0}$ of the crystal lattice if a given device is not a sound absorber, i.e.

1.1 $$\gamma_{0}\ll \gamma_{\mathrm{ph}}\ll \frac{1}{a_{0}}.$$

This condition means that although sound absorption is enhanced by the presence of solid–fluid interfaces, it remains relatively weak and can be considered perturbatively. At low frequencies (in a phononic crystal below the first band gap), sound dispersion is linear, $\omega =c_{\mathrm{eff}}k$, and weak dissipation gives rise to a pure imaginary correction to the wave vector, $k=\omega /c_{\mathrm{eff}}+i\gamma_{\mathrm{ph}}$. In the lowest approximation over viscosity, the effective speed of sound $c_{\mathrm{eff}}$ can be calculated for an inviscid background fluid; see [22].

A sound wave with wavelength 2π/k reflecting from a solid flat wall loses a portion ΔE/E of its energy. Since in the lowest approximation over η and ξ the dissipated energy is concentrated within the boundary layer δ, the energy losses can be estimated as $\Delta E/E∼\delta k/\left(2\pi \right)∼(\sqrt{\omega \eta /\rho })/c_{\mathrm{eff}}$; see e.g. [1,23]. If the sound wave is scattered at a periodic distribution of cylindrical rods, the quantity δk/(2π) acquires a factor $L_{0}/a_{0}$, where $L_{0}$ is the circumference of the cylinders. The relative energy losses within the unit cell become $\Delta E/E∼(\delta k/\left(2\pi \right))(L_{0}/a_{0})$. The decay coefficient $\gamma_{\mathrm{ph}}$ in the exponential factor $e^{-\gamma_{\mathrm{ph}}x}$ is the energy loss per unit length and in this approximation is given by

1.2 $$\gamma_{\mathrm{ph}}=\frac{\Delta E}{a_{0}E}∼\frac{1}{c_{\mathrm{eff}}a_{0}}\sqrt{\frac{f\omega \eta }{\rho }},$$

where $f∼{\left(L_{0}/a_{0}\right)}^{2}$ is the filling fraction of solid cylinders [24]. This qualitative estimate already demonstrates the enhanced absorption of acoustic energy in phononic crystals relative to absorption in free fluid. Note the square-root dependence on the filling fraction. The exact result for $\gamma_{\mathrm{ph}}$ confirms this dependence only at small filling fractions, f≪1.

Equation (1.2) for the decay coefficient is valid by order of magnitude for a simple isotropic lattice and non-interacting scatterers. Anisotropy is a property usually associated with crystalline solids. While natural Newtonian fluids are isotropic, in metamaterials anisotropy can be artificially introduced by inserting scatterers into an isotropic fluid. This results in an anisotropic elastic metafluid [22,25]. An example of extreme anisotropy can be found in the so-called hyperbolic metamaterials [26]. It is clear that in the general case the decay coefficient depends on the direction of propagation of sound. This property means that at low frequencies a solid–fluid phononic crystal behaves like a viscous metafluid with anisotropic viscosity.

In this article, we explore the analytical theory of homogenization developed in [24] for two-dimensional (2D) phononic crystals composed of solid rods in a viscous background to study the effects of strong anisotropy on sound absorption. We consider different 2D phononic crystals with different Bravais lattices and scatterers of different symmetries. We also analyse the behaviour of $\gamma_{\mathrm{ph}}$ at high filling fractions where square-root dependence on f is replaced by a growth of viscous losses. In the limit where the separation between neighbouring rods is comparable to the thickness δ, the growth becomes extremely fast. This effect is of the same nature as the enhanced decay of sound propagating through narrow channels and holes [8–10,12–15,18,20,21].

## 2. Decay coefficient

Derivation of the analytical formula for the decay coefficient $\gamma_{\mathrm{ph}}=\Delta E/(a_{0}E)$ is based on the well-known result for power dissipated around a solid body oscillating in a viscous fluid [1],

2.1 $$\Delta E=\frac{1}{2\sqrt{2}}\sqrt{\rho \omega \eta }\oint_{l_{0}}|v(r){|}^{2}\;dl.$$

This result can be equally applied to calculate viscous losses of a monochromatic sound wave scattered by a motionless solid body. Here, the dissipated power is proportional to $\sqrt{\eta }$, which means that viscous losses occur within the boundary layer. In the principal approximation over viscosity, the velocity field v(r) generated in the fluid by incoming and scattered sound waves is calculated for an inviscid fluid, η=0. The contour of integration $l_{0}$ goes along the circumference of the rod. Note that equation (2.1) becomes meaningless, giving zero losses, if v(r) is the field of velocities in a viscous fluid, where a no-slip boundary condition is applied at any point of the solid–fluid boundary.

It is assumed in equation (2.1) that sound does not penetrate inside a solid body, i.e. the rods of the phononic crystal are considered to be hard scatterers. Equation (2.1) gives the dissipated power within a unit cell of a 2D phononic crystal, assuming that viscous losses in the bulk are neglected. It is valid if $\delta \ll L_{0}$, where $L_{0}$ is the length of the contour of integration $l_{0}$. This inequality, together with the condition of homogenization at low frequencies, defines the frequency interval where the theory is valid,

2.2 $$\frac{\eta }{\rho L_{0}^{2}}\ll \omega \ll \frac{c_{\mathrm{eff}}}{a_{0}}.$$

For solid rods with a circumference of about $L_{0}\geq 1\;\mathrm{mm}$ in a water environment, this inequality is satisfied for frequencies starting from around 1 Hz, i.e. practically it is not a limitation. The upper limit for ω is the lower edge of the band gap. The distribution of velocities v(r) caused by the propagation of sound can be calculated by expansion of the wave equation over plane waves. Using the same velocity field v(r), the acoustic energy can be calculated by integration over the unit cell. In the low-frequency limit, ω,k→0, the following result was obtained for the decay coefficient $\gamma_{\mathrm{ph}}=\Delta E/(a_{0}E)$ [24]:

2.3 $$\gamma_{\mathrm{ph}}(\hat{k})=\frac{L_{0}}{2A_{c}c_{\mathrm{eff}}(\hat{k})}\sqrt{\frac{\omega \eta }{2\rho }}\frac{M(\hat{k})}{N(\hat{k})}.$$

Here, $\hat{k}=k/k$ is the unit vector in the direction of propagation, $A_{c}$ is the area of the unit cell, and the functions $M(\hat{k})$ and $N(\hat{k})$ account for integration over the contour $l_{0}$ and over the area of the unit cell $A_{c}$, respectively. They strongly depend on the geometry of the unit cell, the distribution of mass density within it and the direction of propagation. These two terms can be represented by the following series over the reciprocal lattice vectors G:

2.4 $$\begin{array}{rl} M(\hat{k}) & \;=1+\frac{2}{\rho }\sum_{G,G^{\prime }}L^{*}(G)(\hat{k}\cdot G)(\hat{k}\cdot G^{\prime })F(G^{\prime })I(G,G^{\prime })\\ & \;\;+\frac{1}{\rho^{2}}\sum_{G_{1},\ldots ,G_{4}}L^{*}(G_{1}+G_{3})(G_{1}\cdot G_{3})(\hat{k}\cdot G_{2})(\hat{k}\cdot G_{4})\\ & \;\;\times F(G_{2})F(G_{4})I(G_{1},G_{2})I(G_{3},G_{4}) \end{array}$$

and

2.5 $$\begin{array}{rl} N(\hat{k}) & \;=1-f-\frac{2}{\rho }\sum_{G,G^{\prime }}\hat{k}\cdot G\;\hat{k}\cdot G^{\prime }F^{*}(G)F(G^{\prime })I(G,G^{\prime })\\ & \;\;-\frac{1}{\rho^{2}}\sum_{G_{1},\ldots ,G_{4}}F^{*}(G_{1}+G_{3})(G_{1}\cdot G_{3})(\hat{k}\cdot G_{2})(\hat{k}\cdot G_{4})\\ & \;\;\times F(G_{2})F(G_{4})I(G_{1},G_{2})I(G_{3},G_{4}). \end{array}$$

Here, $I(G,G^{\prime })={\left[G\cdot G^{\prime }\nu (G-G^{\prime })\right]}^{-1}$, ν(G) is the Fourier component of the periodic function 1/ρ(r), and the linear form-factor L(G) is defined as the integral over the circumference of the solid scatterer,

2.6 $$L(G)=\frac{1}{L_{0}}\oint_{l_{0}}\;e^{-iG\cdot r}\;dl.$$

The effective speed of sound $c_{\mathrm{eff}}(\hat{k})=\lim_{k\to 0}(\omega /k)$ was calculated in [22] and is explicitly given by

2.7 $$\begin{array}{rl} & c_{\mathrm{eff}}^{2}(\hat{k})=\frac{1}{\bar{\beta }}\left[\bar{\nu }-\sum_{{G,G}^{\prime }\neq 0}(\hat{k}\cdot G)\;(\hat{k}\cdot G^{\prime })\nu (G)\nu (-G^{\prime })I(G^{\prime },G)\right]\\ & \text{with}\;\bar{\nu }=\frac{f}{\rho_{s}}+\frac{1-f}{\rho }\;\text{and}\;\bar{\beta }=\frac{f}{\lambda_{s}}+\frac{1-f}{\lambda }, \end{array}$$

where $\lambda_{s}$ and $\rho_{s}$ are the bulk modulus and density of the rods, respectively. In the approximation of hard scatterers, $\lambda_{s}\gg \lambda$ and $\rho_{s}\gg \rho$.

## 3. Results and discussion

Using the equations (2.3)–(2.7), we calculated the decay coefficient as a function of the filling fraction and the direction of propagation for square and hexagonal lattices with period $a_{0}=5.5\;\mathrm{mm}$. Calculations were performed for a sound frequency of ω/(2π)=50 kHz, which lies well below the band gap of the corresponding structures. Since $\gamma_{\mathrm{ph}}∼\sqrt{\omega }$, the decay coefficient can be rescaled to any other frequency within the first transmission band where the dispersion is linear but anisotropic, $\omega =c_{\mathrm{eff}}(\hat{k})k$. Water is taken as the background fluid. To increase the effects of anisotropy, for each of the 2D lattices mentioned earlier we considered solid rods with square, rectangular and triangular cross-sections. To reach the limit of hard scatterers, the elastic parameters of the rods, $\lambda_{s}$ and $\rho_{s}$, were selected to be $10^{3}$ times those of aluminium. The thickness δ of the viscous layer in water is approximately 3 μm at frequency 50 kHz.

**Figure 1.**
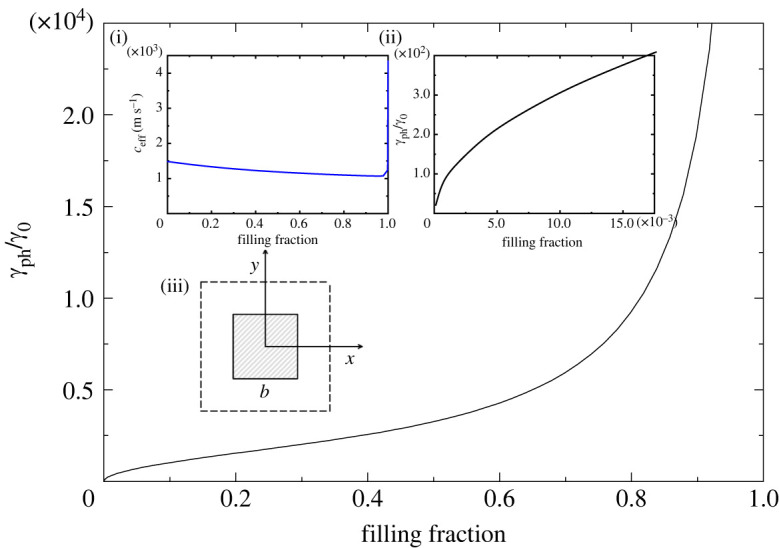
Normalized decay coefficient of sound in a square lattice with square cross-section of the rods in a viscous water background as a function of the filling fraction. Insets show (i) the effective speed of sound as a function of the filling fraction; (ii) blow-up of the interval for small values of f, where $\gamma_{\mathrm{ph}}∼\sqrt{f}$; (iii) the unit cell. Owing to the fourfold rotational symmetry, the decay coefficient and the effective speed of sound are isotropic. (Online version in colour.)

**Figure 2.**
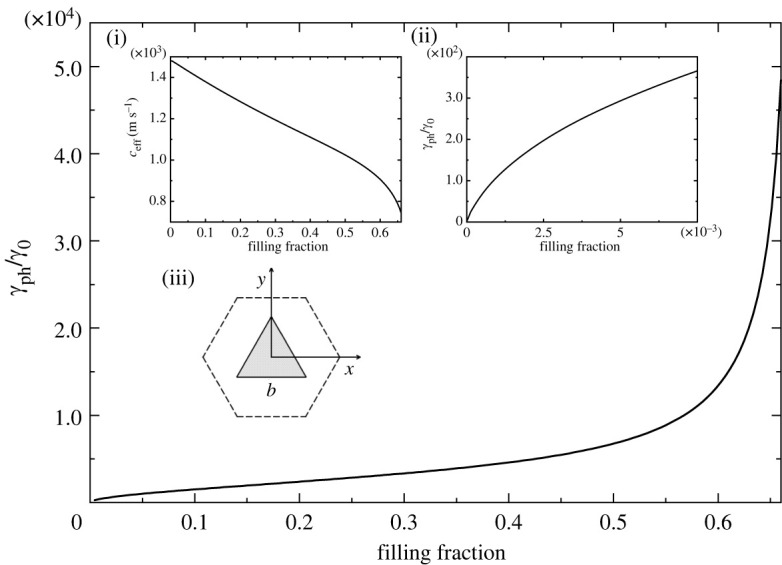
Normalized decay coefficient of sound in a hexagonal lattice with equilateral triangular rods of side length b as a function of the filling fraction. The limit of touching scatterers is reached at $f_{\max}=2/3$. Insets show (i) the effective speed of sound as a function of the filling fraction; (ii) blow-up of the interval for small values of f, where $\gamma_{\mathrm{ph}}∼\sqrt{f}$; (iii) the unit cell. Owing to the threefold rotational symmetry, the decay coefficient and the effective speed of sound are isotropic.

**Figure 3.**
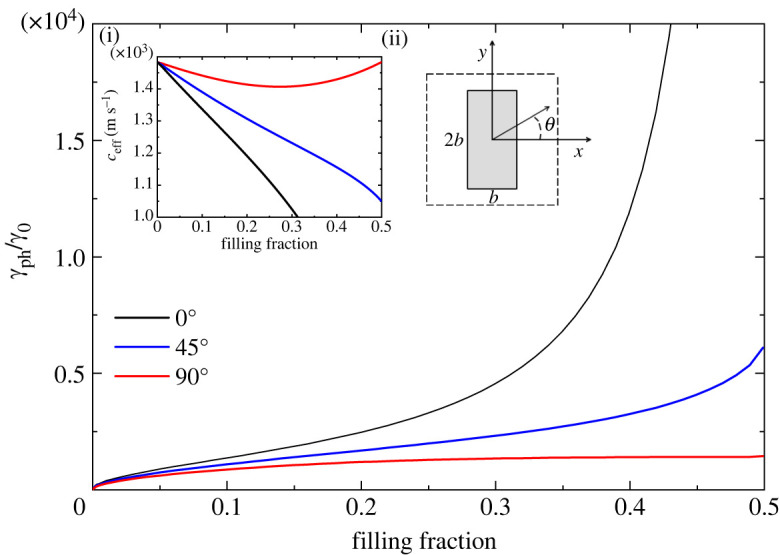
Normalized decay coefficient of sound in a square lattice with rectangular rods in water as a function of the filling fraction, for three different directions of propagation, $\theta =0^{\circ },\;45^{\circ }\;\text{and}\;90^{\circ }$. The limit of touching scatterers is reached at $f_{\max}=0.5$. Insets show (i) the anisotropic effective speed of sound as a function of the filling fraction and (ii) a schematic representation of the unit cell (where the ratio of side lengths of the rectangular rods is 2 : 1). (Online version in colour.)

**Figure 4.**
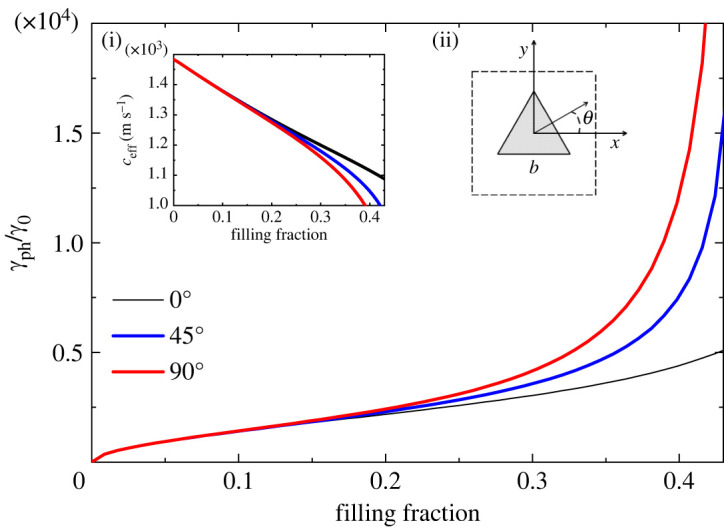
Normalized decay coefficient of sound in a square lattice with equilateral triangular cross-sections of the rods of side length b in water as a function of the filling fraction, for three different directions of propagation, $\theta =0^{\circ },\;45^{\circ }\;\text{and}\;90^{\circ }$. The limit of touching scatterers is reached at $f_{\max}=\sqrt{3}/4=0.433$. Insets show (i) the anisotropic effective speed of sound as a function of the filling fraction and (ii) a schematic representation of the unit cell. (Online version in colour.)

**Figure 5.**
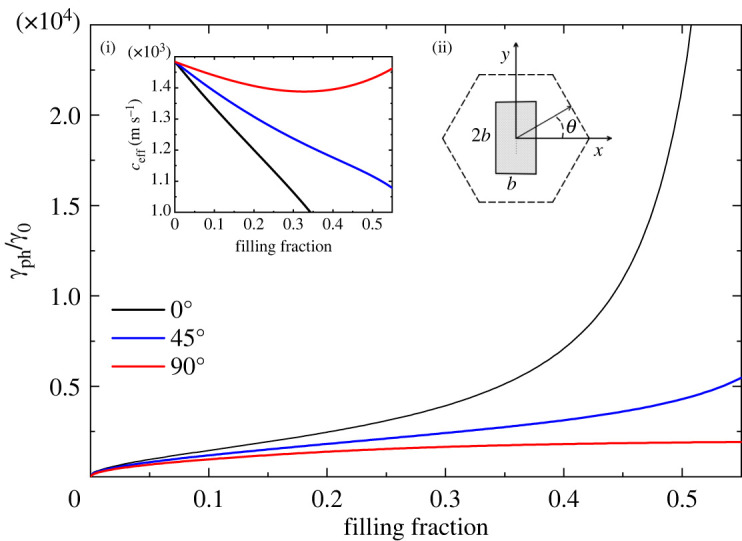
Normalized decay coefficient of sound in a hexagonal lattice with vertical rectangular rods in water as a function of the filling fraction, for three different directions of propagation, $\theta =0^{\circ },\;45^{\circ }\;\text{and}\;90^{\circ }$. The limit of touching scatterers is reached at $f_{\max}=1/\sqrt{3}=0.577$. Insets show (i) the anisotropic effective speed of sound as a function of the filling fraction and (ii) the unit cell. (Online version in colour.)

In figures 1–5, we plot the normalized (with respect to bulk water) decay coefficient $\gamma_{\mathrm{ph}}/\gamma_{0}$ as a function of the filling fraction f for different crystal lattices and different cross-sections of the rods.

Figure 1 and 2 are for square and hexagonal lattices with square and equilateral triangular scatterers, respectively. For artificial periodic structures, the symmetry of the unit cell is determined by both the symmetry of the Bravais lattice and the symmetry of the scatterers. Scatterers in a phononic crystal may have lower symmetry than the Bravais lattice.

The effective speed of sound in equation (2.7) can be represented as a product $c_{\mathrm{eff}}=A_{ik}{\hat{k}}_{i}{\hat{k}}_{k}$ of the second-rank tensor $A_{ik}$ and the diad ${\hat{k}}_{i}{\hat{k}}_{k}$ [22]. The decay coefficient equations (2.3)–(2.5) also can be written in terms of two second-rank tensors, $\gamma_{\mathrm{ph}}(\hat{k})=B_{ik}{\hat{k}}_{i}{\hat{k}}_{k}/(C_{lm}{\hat{k}}_{l}{\hat{k}}_{m})$. These three tensors $A_{ik}$, $B_{ik}$ and $C_{ik}$ define the parameters of anisotropy of elastic and dissipative 2D phononic crystals.

It is known that in a crystal lattice possessing a third- or higher-order rotational axis of symmetry, a second-rank symmetric tensor is reduced to a scalar. The structures depicted in figures 1 and 2 possess a fourth- and third-order rotational axis, respectively. Therefore, the effective speed of sound and the decay coefficient do not depend on the direction of propagation, i.e. these metamaterials are isotropic in the homogenization limit. There are three insets in each of figures 1 and 2. Inset (i) shows the effective speed of sound as a function of the filling fraction. For the square lattice with square rods, the effective speed of sound is presented for the full range of allowable values of the filling fraction ($0\leq f\leq f_{\max}=1$). We observe that the speed of sound gradually decreases with f, and there is a sharp turn near f=1 towards the value $\sqrt{\lambda_{\mathrm{Al}}/\rho_{\mathrm{Al}}}=4346\;{m\;s}^{-1}$, which is the speed of longitudinal sound in aluminium. Although we present the effective speed of sound for the full range of possible filling fractions, the sound decay constant is not calculated for values very close to $f=f_{\max}=1$, since the maximum permitted value of filling fraction must be slightly less than the limit of touching scatterers to prevent overlapping of two viscous layers formed around the neighbouring scatterers.

Inset (ii) shows the region of small filling fractions. For all the structures considered in this work, the decay coefficient grows as $\gamma_{\mathrm{ph}}=\sqrt{f}$ at low filling fractions, as predicted by the qualitative result presented in equation (1.2). In figures 1 and 2, the decay coefficient is plotted specifically for f≪1, where the square-root dependence is clearly observed. Finally, inset (iii) is a schematic representation of the unit cell. As can be seen in both figures, within the region of filling fractions corresponding to practical applications of phononic crystals, 0<f<0.6, the decay length of sound is reduced by a factor of $10^{3}\text{–}10^{4}$ from that in bulk water

For less symmetric structures, the effective parameters $c_{\mathrm{eff}}$ and $\gamma_{\mathrm{ph}}$ depend on the angle θ characterizing the direction of propagation $\hat{k}=(\cos\theta ,\sin\theta )$ of the sound wave, and for these configurations the homogenized phononic crystal behaves like an anisotropic viscous fluid. In figures 3 and 4, we present the normalized decay coefficient for three different directions of propagation as a function of the filling fraction for a square lattice with rectangular and equilateral triangular rods, respectively. The black, blue and red lines correspond to the directions of propagation $\theta =0^{\circ },\;45^{\circ }\;\text{and}\;90^{\circ },\;\text{respectively}$. For this lattice, the metamaterial with rectangular cross-section of the rods exhibits the highest anisotropy, as can be seen by comparing the values of the decay coefficient in the two figures at f=0.4. The highest value for the decay coefficient is obtained for $\theta =0^{\circ }$. Sound propagating along this direction is transmitted through a narrow viscous channel between two scatterers. It is easy to analyse the role of the viscous layer within a narrow channel for the anisotropic unit cell of a square lattice with rectangular inclusions oriented by its longer side along the y-axis. There are two viscous layers of width δ=2.52 μm occupying only 0.13% of the spacing between the scatterers for a filling fraction of 0.499. While the viscous layers occupy a negligible volume of the channel, the decay of sound is relatively high ($\gamma_{\mathrm{ph}}/\gamma_{0}∼10^{4}$). Furthermore, a similar situation occurs for triangular inclusions; see figure 4. In both cases, we have the formation of slit cavities where the velocity gradients reach very high values.

Finally, in figure 5 we present the normalized decay coefficient for the hexagonal lattice with rectangular rods. For this metamaterial, the maximum value of the decay coefficient is obtained for a sound wave propagating along the direction $\theta =0^{\circ }$. While for this direction sound dissipates its energy along the shorter side of the rectangle, the energy losses reach their maximum because the wave frequently meets the corners. Near each corner the velocity field exhibits a complicated pattern with very high values of its gradients. Apparently, dissipation near sharp corners makes a considerable contribution that leads to a maximum of wave attenuation.

The effects of enhanced acoustic dissipation in narrow slit cavities have been recently studied in [8,14]. For example, in [8] the authors observed a 5% reduction in the speed of sound propagating in a narrow air cavity slit in an aluminium matrix. They attribute this effect to dissipation, which occurs within the viscous boundary layers occupying only 5% of the total slit width. In [14], a 28% reduction in the transmission of sound is found when the thermoviscous boundary layer thickness is only about 2.3% of the width of the slit cut in an acoustic metasurface.

**Figure 6.**
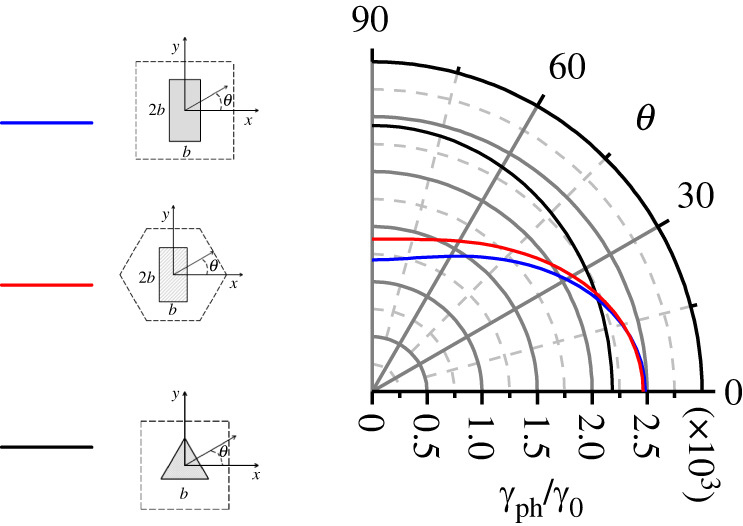
Normalized decay coefficient of sound for anisotropic unit cells for a 30% filling fraction as a function of the angle θ. Insets show a square lattice with rectangular rods oriented by its longer side along the *y*-direction, a hexagonal lattice with rectangular rods and a square lattice with equilateral triangular rods. (Online version in colour.)

An alternative way to analyse the anisotropic behaviour is depicted in figure 6. Here, we plot a polar diagram showing the angular dependence of the decay coefficient for three different lattices with different scatterers but the same filling fraction, f=30%. The three curves in figure 6 are ellipses with semiaxes representing maximum and minimum attenuation of sound along the corresponding directions. Note that elliptical angular dependence of the speed of light in 2D photonic crystals was previously reported in [27]. The plots in figure 6 demonstrate that viscous losses strongly depend on the shape of the scatterers. The main factors that define viscous attenuation are the narrow channels between neighbouring scatterers, the number of corners per unit length and the sharpness of these corners. The combination of these factors leads to a complicated anisotropic pattern of viscous losses, which is impossible to analyse using only qualitative or phenomenological approaches.

## 4. Conclusion

In conclusion, we have presented a possible way of tuning the decay coefficient of sound in a phononic crystal with a viscous background. A periodic set of solid rods in a viscous fluid homogenizes in the low-frequency limit and behaves for sound waves like an anisotropic viscous fluid. The level of anisotropy of the decay coefficient can be tuned to the desired value by selecting appropriately the crystal lattice, cross-section of the rods and filling fraction of the rods. We predict strong enhancement of sound decay along the directions where sound propagates through narrow slits formed by neighbouring scatterers.

## Data Availability

This article has no additional data.
